# Genetic Deletion of Soluble Epoxide Hydrolase Attenuates Inflammation and Fibrosis in Experimental Obstructive Nephropathy

**DOI:** 10.1155/2015/693260

**Published:** 2015-01-22

**Authors:** Chin-Wei Chiang, Hsueh-Te Lee, Der-Cherng Tarng, Ko-Lin Kuo, Li-Ching Cheng, Tzong-Shyuan Lee

**Affiliations:** ^1^Department of Physiology, National Yang-Ming University, Taipei 11221, Taiwan; ^2^Institute of Anatomy and Cell Biology, National Yang-Ming University, Taipei 11221, Taiwan; ^3^Division of Nephrology, Department of Medicine and Immunology Research Centre, Taipei Veterans General Hospital, Taipei 11221, Taiwan; ^4^Division of Nephrology, Buddhist Tzu Chi General Hospital, Taipei Branch, Taipei 23142, Taiwan; ^5^Department of Nursing, Chang Gung Institute of Technology, Taoyuan 33303, Taiwan

## Abstract

Soluble epoxide hydrolase (sEH) is abundantly expressed in kidney and plays a potent role in regulating inflammatory response in inflammatory diseases. However, the role of sEH in progression of chronic kidney diseases such as obstructive nephropathy is still elusive. In current study, wild-type (WT) and* sEH* deficient (*sEH* ^−/−^) mice were subjected to the unilateral ureteral obstruction (UUO) surgery and the kidney injury was evaluated by histological examination, western blotting, and ELISA. The protein level of sEH in kidney was increased in UUO-treated mice group compared to nonobstructed group. Additionally, UUO-induced hydronephrosis, renal tubular injury, inflammation, and fibrosis were ameliorated in* sEH* ^−/−^ mice with the exception of glomerulosclerosis. Moreover,* sEH* ^−/−^ mice with UUO showed lower levels of inflammation-related and fibrosis-related protein such as monocyte chemoattractant protein-1, macrophage inflammatory protein-2, interleukin-1*β* (IL-1*β*), IL-6, inducible nitric oxide synthase, collagen 1A1, and *α*-actin. The levels of superoxide anion radical and hydrogen peroxide as well as NADPH oxidase activity were also decreased in UUO kidneys of* sEH* ^−/−^ mice compared to that observed in WT mice. Collectively, our findings suggest that sEH plays an important role in the pathogenesis of experimental obstructive nephropathy and may be a therapeutic target for the treatment of obstructive nephropathy-related diseases.

## 1. Introduction

Urinary obstruction, defined as functional or anatomic impedance to the flow of urine along the urinary tract leading to hydronephrosis and declining renal function, is a common cause of acute and chronic renal failure [1]. It can be caused by renal calculi, renal tumor, or ureter malformation in newborn and results in difficulty in micturition with subsequent kidney injury [2]. Progression of renal dysfunction in obstructive nephropathy is associated with acute renal tubular injury and inflammatory response. In addition, chronic obstructive nephropathy causes resident fibroblast activation which leads to renal interstitial fibrosis and glomerulosclerosis [3, 4]. The extent of renal impairment eventually results in the development of chronic kidney disease [5]. Despite the fact that several predisposing factors to the development of urinary obstruction have been identified [6], the detailed molecular mechanisms underlying the pathogenesis of obstructive nephropathy are not fully understood.

Soluble epoxide hydrolase (sEH), a key enzyme responsible for the conversion of epoxyeicosatrienoic acids (EETs) to the corresponding dihydroxyeicosatrienoic acids (DHETs), is widely distributed in mammalian tissues, including kidney, liver, and heart [7, 8]. Pharmacological inhibition of the hydrolase activity and genetic disruption of* sEH* both can increase the accumulation of EETs and other epoxy fatty acids which attenuate angiotensin II-induced hypertension and cardiac hypertrophy and fibrosis [9, 10]. Furthermore, inhibition of sEH has been shown to exert protective effects in diabetes-induced renal injury [11]. These lines of evidence strongly suggest that sEH may be a therapeutic target for hypertension-induced complications. Even though important progression has been made in the therapeutic value of sEH inhibition [12], the participation of sEH in the pathogenesis of chronic obstructive nephropathy and the molecular mechanisms it involved require further investigation.

In the present study, we aimed to address the potential role and underlying mechanism of sEH in pathogenesis of obstructive nephropathy by using a unilateral ureteral obstruction (UUO) mouse model, a well-known model for obstructive nephropathy [13]. First, we examined the expression profile of sEH in the development of experimental obstructive nephropathy. Second, we aimed to assess the effect of genetic deletion of* sEH* on UUO-induced inflammation, fibrosis, and glomerulosclerosis in kidneys. Our results showed that genetic deletion of* sEH* attenuated UUO-induced hydronephrosis, renal tubular injury, inflammatory response, collagen deposition, and fibrosis in mice. These findings indicate that inhibition of sEH may be a novel therapeutic strategy for treatment of obstructive nephropathy-related diseases.

## 2. Materials and Methods

### 2.1. Reagents and Assay Kits

Rabbit anti-collagen (COL)4A2, anti-sEH, goat anti-COL1A1, mouse anti-*α*-actin, and rat anti-CD3 antibodies were from Santa Cruz Biotechnology (Santa Cruz, CA, USA). Rabbit anti-inducible nitric oxide synthase (iNOS) and anti-myeloperoxidase (MPO) antibodies were from Cell Signaling Technology (Beverly, MA, USA). Rat anti-F4/80 antibody was from Abcam (Cambridge, MA, USA). Mouse anti-GAPDH antibody and Masson's trichrome staining kits were from Sigma-Aldrich (St. Louis, MO, USA). Periodic acid-Schiff staining kit was from Muto Pure Chemical (Tokyo, Japan). Enzyme-linked immunosorbent assay (ELISA) kits for monocyte chemotactic protein-1 (MCP-1), macrophage inflammatory protein-2 (MIP-2), interleukin- (IL-) 1*β*, and IL-6 were from R&D Systems (Minneapolis, MN, USA). Quantitative assay kit for collagen Sirius Red Staining was from Amsbio (Lake Forrest, CA, USA). Hydroethidine (DHE) and 2′,7′-dichlorofluorescin diacetate (DCFH-DA) were from Molecular Probes (Eugene, OR, USA). EnzyChrom NADP^+^/NAD(P)H assay kit was from BioAssay Systems (Hayward, CA, USA).

### 2.2. Unilateral Ureteral Obstruction Mouse Model

The investigation conformed to the* Guide for the Care and Use of Laboratory Animals* published by US National Institutes of Health (NIH Publication number 82-23, revised 1996), and all animal experiments were approved by the Animal Care and Utilization Committee of the National Yang-Ming University. Male WT C57BL/6 mice were from the National Laboratory Animal Center, National Science Council (Taipei, Taiwan);* Ephhx2* ^*tm1/Gon2/J*^ (*sEH* ^−/−^) mice were from Jackson Laboratory (Bar Harbor, ME, USA). Mice were housed in barrier facilities on a 12 h light/12 h dark cycle and fed with normal chow diet. Eight- to ten-week-old male WT and *sEH* ^−/−^ C57BL/6 mice were anesthetized with pentobarbital (80 mg/kg, intraperitoneally) and the left kidney was exposed through an incision at the dorsal midline skin. Then, the connective tissues that surrounded the kidney were cleared to expose the ureter. Next, the ureter was ligated by two independent 4–0 Nylon sutures. The muscle and skin were closed by 5–0 Ethilon sutures. The right kidney was used as nonobstructed group, which is under the same operation but without ureter ligation. At the end of experiment, mice were euthanized by CO_2_ and then kidneys were harvested and separated into two parts: one part of kidney was fixed with 4% paraformaldehyde, embedded in paraffin for histological analysis; another part of kidney was stored at −80°C for further analysis.

### 2.3. Immunohistochemical Assessment

Kidney sections were reacted with 3% H_2_O_2_ for 10 min. After blocking with 1% bovine serum albumin for 1 hour, samples were incubated with anti-sEH antibody overnight at 4°C and then with corresponding secondary antibody for 2 h at 37°C. Antigenic sites were visualized by adding 3,3′-diaminobenzidine and hematoxylin was used for counterstaining.

### 2.4. Masson's Trichrome Staining

Kidney sections were treated with Bouin's solution overnight. After being washed with PBS, samples were stained by Weigert's iron hematoxylin and Biebrich Scarlet-acid Fuchsin solution. These sections were reacted with phosphomolybdic-phosphotungstic acid solution and then stained with aniline blue.

### 2.5. Quantitative Assessment of Collagen with Sirius Red Staining

Briefly, deparaffinized sections were incubated with dye solution (0.1% Sirius Red and 0.1% Fast Green in periodic acid solution) (Polysciences) at room temperature for 30 min. The color was then eluted from the sections by incubation with 0.05 M NaOH in methanol and measured at OD 540 nm and OD 605 nm by spectrophotometer. The amount of collagen in tissue sections was calculated according to the manufacturer's instructions (collagen (*μ*g/section) = [OD540 − (OD605 × 0.291)]/37.8 × 1000).

### 2.6. Measurement of Inflammatory Cytokines

The concentrations of proinflammatory cytokines in kidney including MCP-1, MIP-2, IL-1*β*, and IL-6 were measured by use of ELISA kits.

### 2.7. Periodic Acid-Schiff Staining

Periodic acid-Schiff staining was performed according to the manufacturer's instructions. Briefly, sections were deparaffinized and reacted with 1% periodic acid solution. The samples were then reacted with Schiff's reagent and washed by NaHSO_3_/HCl solution. Hematoxylin was used for counterstaining.

### 2.8. Microscopy Assessment of Histological Changes in UUO-Induced Renal Injury

Kidney section slides were deparaffinized and stained with hematoxylin and eosin Y (H&E) and then viewed under a Motic TYPE 102M microscope. The scoring of microscopy assessments of tubular dilation, atrophy, leukocyte infiltration, and tubular interstitial volume and glomerulosclerosis was summarized in Tables 1 and 2.

### 2.9. Western Blot Analysis

Tissues were lysed with phosphate buffered saline containing 1% Triton X-100, 0.1% SDS, 0.5% sodium deoxycholate, 1 *μ*g/mL leupeptin, 10 *μ*g/mL aprotinin, 1 mM PMSF, Tyr phosphatase cocktail I, and Ser/Thr phosphatase cocktail II on ice. After sonication, tissue extracts underwent centrifugation at 12000 ×g for 5 min at 4°C. The supernatants were collected as tissue lysates. All protein concentrations were examined by Bradford protein-binding assay. Aliquots (50 *μ*g) of lysates were separated on 8% or 12% SDS-PAGE and then transblotted on an Immobilon-P membrane (Millipore, Bedford, MA). After being blocked with 5% skim milk for 1 h, blots were incubated with primary antibodies and then with corresponding secondary antibodies. The protein bands were detected by use of an enhanced chemiluminescence kit (PerkinElmer, Boston, MA) and quantified by ImageQuant 5.2 software (Healthcare Bio-Sciences, PA).

### 2.10. Quantification of Tissue Reactive Oxygen Species (ROS) Production

The measurement of superoxide anion radical (O_2_^−^) and hydrogen peroxide (H_2_O_2_) counts was performed as previously described with minor modification [14]. Briefly, kidneys were homogenized in 0.2 mL of PBS (pH 7.4) and further diluted with the same buffer and placed on ice. Kidney homogenates (100 *μ*g) were incubated in PBS containing 10 *μ*M DHE or 20 *μ*M DCFH-DA at 37°C for 60 min and the fluorescence intensity of the lysates was analyzed by use of a multilabel counter (PerkinElmer, Waltham, MA) at 530 nm excitation and 620 nm emission for ETH and at 488 nm excitation and 530 nm emission for DCF.

### 2.11. Measurement of NADPH Oxidase Activity

The activity of NADPH oxidase in kidney homogenates (100 *μ*g) was analyzed by EnzyChrom NADP^+^/NADPH assay kit according to the manufacturer's instructions.

### 2.12. Statistical Analysis

Data are presented as mean ± SEM. Mann-Whitney test was used to compare 2 independent groups and Kruskal-Wallis test followed by Bonferroni post hoc analysis for multiple groups. SPSS v20.0 (SPSS Inc., Chicago, IL) was used for analysis. Differences were considered statistically significant at *P* < 0.05.

## 3. Results

### 3.1. Expression of sEH Is Increased in UUO-Induced Obstructive Nephropathy

**Figure 1:**
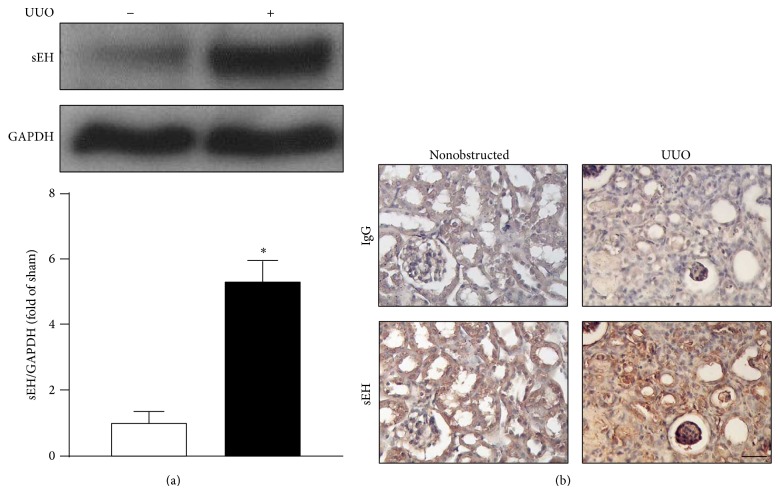
Expression of sEH in kidney is increased after unilateral ureteral obstruction (UUO) surgery. (a) Eight-week-old WT mice received UUO surgery. In 14 days after UUO, kidneys were harvested and tissue lysates were subjected to western blot to examine the protein levels of sEH and GAPDH. (b) Kidney specimens from mice were immunostained with anti-IgG or anti-sEH antibody and then recognized by corresponding horseradish peroxidase-conjugated secondary antibody. Hematoxylin was used for counterstaining. Scalar bar: 50 *μ*m. Data are mean ± SEM from 8 mice. ^*^ *P* < 0.05 versus WT nonobstructed kidney.

To explore the possibility of sEH participating in the pathogenesis of obstructive nephropathy, we first investigated the expression of sEH in mouse kidneys under nonobstructed condition and UUO-induced obstructive nephropathy. We found that the protein expression of sEH was increased in UUO kidneys as revealed by western blots (Figure 1(a)). Results of immunohistochemistry showed sEH primarily expressed in renal tubular cells in nonobstructed kidneys (Figure 1(b)). However, expression of sEH was found mainly in interstitial cells and infiltrated leukocytes of UUO kidneys (Figure 1(b)). Thus, our results suggest that sEH may play an important role in development of obstructive nephropathy.

### 3.2. Knockout of sEH Ameliorates the Hydronephrosis and Renal Tubular Injury

**Figure 2:**
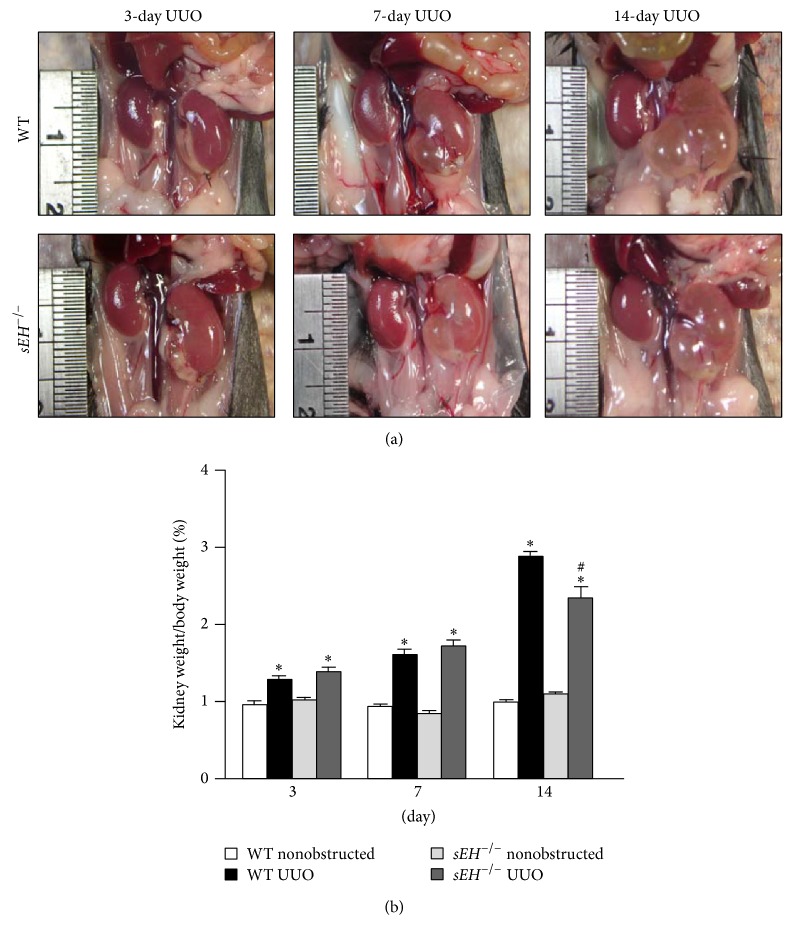
Genetic deletion of* sEH* ameliorates the UUO-induced hydronephrosis. (a) At 3, 7, and 14 days after UUO surgery, mice were euthanized by CO_2_ and kidneys were photographed. (b) The ratio of kidney weight and body weight. Data are mean ± SEM from 8 mice. ^*^ *P* < 0.05 versus WT nonobstructed kidney; ^#^ *P* < 0.05 versus WT UUO kidney.

**Figure 3:**
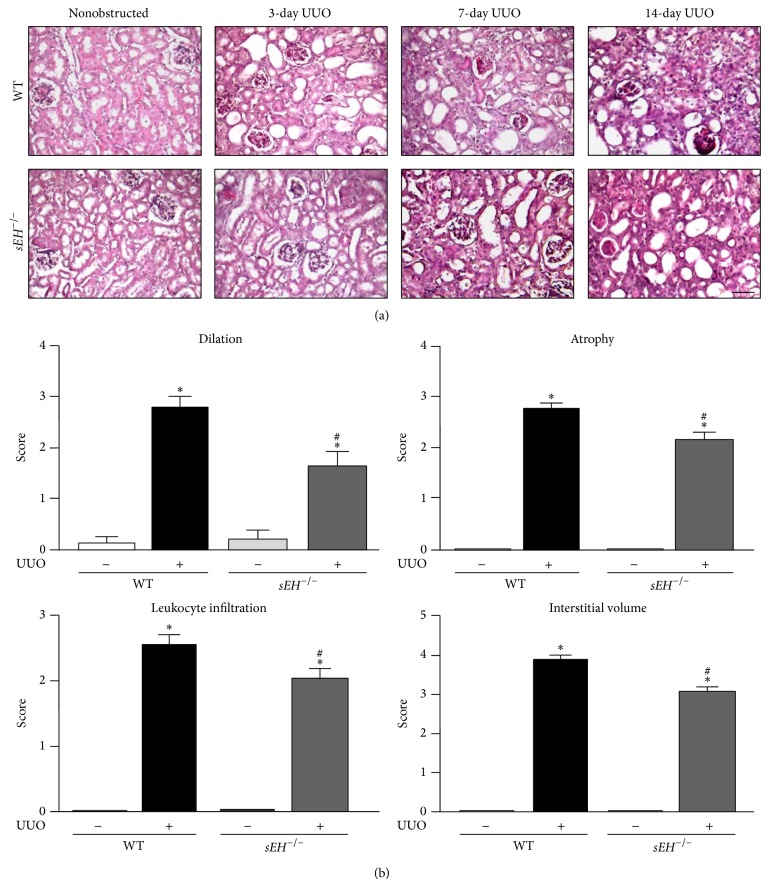
Genetic deletion of* sEH* attenuates the UUO-induced obstructive nephropathy. (a) Kidney specimens from WT and *sEH* ^−/−^ mice were stained with hematoxylin and eosin Y. Scale bar: 100 *μ*m. (b) Quantification of histopathology of kidney: renal tubular dilation, renal tubular atrophy, leukocyte infiltration, and interstitial volume. Data are mean ± SEM from 8 mice. ^*^ *P* < 0.05 versus WT nonobstructed kidney; ^#^ *P* < 0.05 versus WT UUO kidney.

Next, we used a loss-of-function strategy to delineate the potential role of sEH in the development of UUO-induced obstructive nephropathy. Genetic deletion of* sEH* in mice significantly decreased UUO-induced hydronephrosis (Figure 2(a)). The ratios of kidney weight and body weight were also decreased in* sEH* ^−/−^ mice 14 days after UUO surgery (Figure 2(b)). Furthermore, the histological changes in UUO-induced renal tubular injury were attenuated in* sEH* ^−/−^ kidneys as compared with WT kidneys (Figure 3). Tubular dilation, tubular atrophy, leukocyte infiltration, and interstitial volume were all alleviated in UUO-treated* sEH* ^−/−^ kidneys in comparison with WT kidneys (Figure 3). These results suggest that inhibition of sEH may provide protective effects against UUO-induced kidney injury.

### 3.3. Ablation of sEH Reduces Inflammatory Response Elicited by Urinary Obstruction

**Figure 4:**
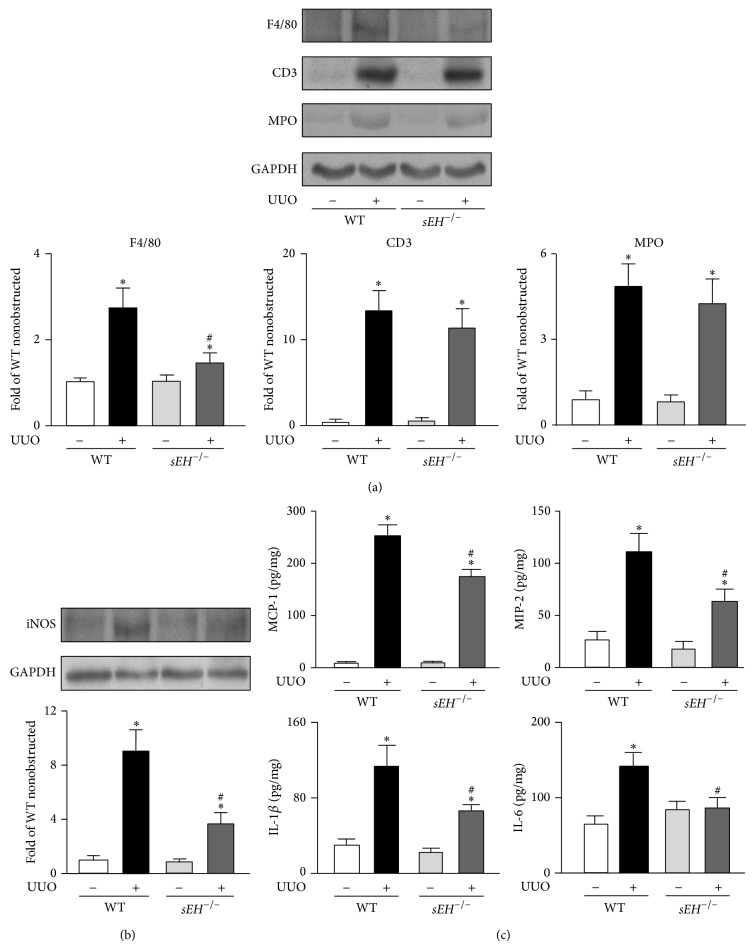
Deficiency of* sEH* reduces the leukocyte infiltration and cytokine production. ((a) and (b)) 14 days after UUO surgery, kidneys were harvested. Kidney lysates from WT and* sEH* ^−/−^ mice were immunoblotted with F4/80, CD3, MPO, inducible nitric oxide synthase (iNOS), and GAPDH. (c) The levels of MCP-1, MIP-2, IL-1*β*, and IL-6 in kidneys were assessed by ELISA kits. Data are mean ± SEM from 8 mice. ^*^ *P* < 0.05 versus WT nonobstructed kidney; ^#^ *P* < 0.05 versus WT UUO kidney.

Infiltration of inflammatory cells is a central event in progression of renal injury through mediating tissue remodeling [15]. We further evaluated the involvement of sEH in regulating inflammatory response in UUO kidneys. Our results showed that, after UUO surgery, the infiltration of macrophages but not neutrophils and T cells was decreased in kidneys of *sEH* ^−/−^ mice as compared with WT mice (Figure 4(a)). Moreover, we investigated the levels of iNOS by western blotting and proinflammatory cytokines including MCP-1, MIP-2, IL-1*β*, and IL-6 by ELISA; all were reduced in *sEH* ^−/−^ mice compared to WT mice (Figures 4(b) and 4(c)). Thus, sEH involvement in the regulation of inflammatory response may be crucial during the development of UUO-induced obstructive nephropathy.

### 3.4. Deletion of sEH Decreases the Interstitial Fibrosis but Has No Significant Effects on Glomerulosclerosis

**Figure 5:**
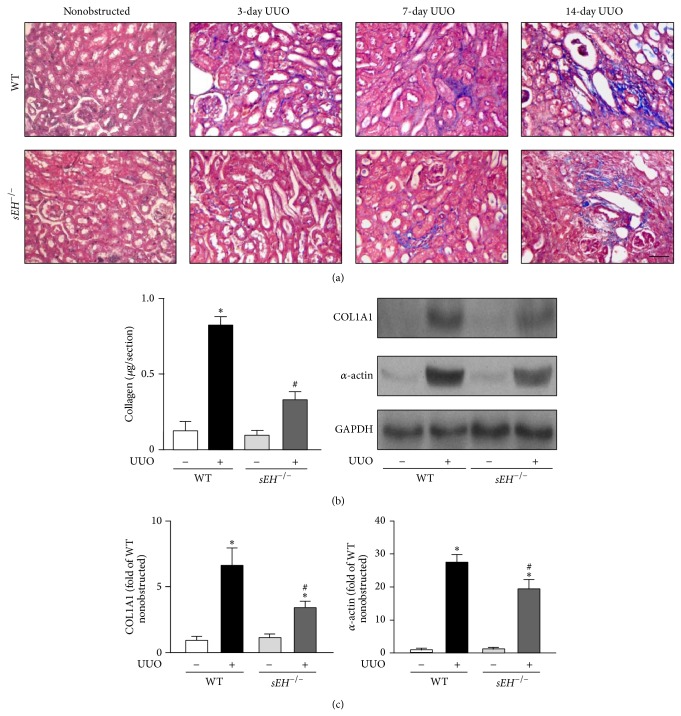
Knockout of* sEH* decreases the UUO-induced collagen deposition and expression of fibrosis-related proteins. ((a) and (b)) 14 days after UUO surgery, kidneys were harvested. Kidney sections from WT and* sEH* ^−/−^ mice were subjected to Masson's trichrome staining or Sirius Red Staining. Scale bar: 100 *μ*m. (c) Kidney lysates were immunoblotted with collagen 1A1 (COL1A1), *α*-actin, and GAPDH. Data are mean ± SEM from 8 mice. ^*^ *P* < 0.05 versus WT nonobstructed kidney; ^#^ *P* < 0.05 versus WT UUO kidney.

**Figure 6:**
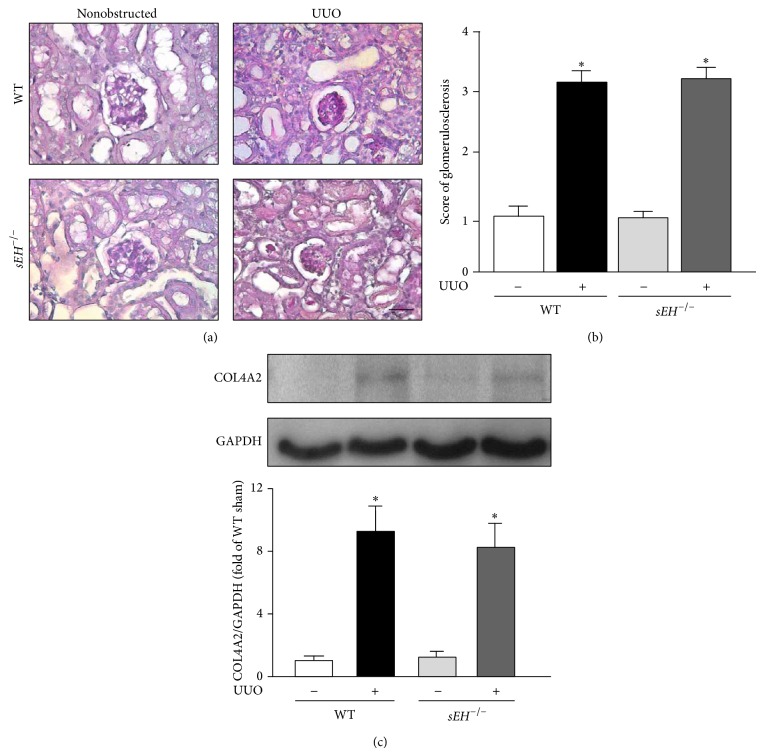
Deletion of* sEH* does not affect the glomerulosclerosis induced by UUO surgery. (a) 14 days after UUO surgery, kidney specimens from WT and *sEH* ^−/−^ mice were subjected to periodic acid-Schiff staining. Hematoxylin was used for counterstaining. Scale bar: 50 *μ*m. (b) Quantification of histopathology of glomerulosclerosis of kidney. (c) Kidney lysates were immunoblotted with collagen 4A2 (COL4A2) and GAPDH. Data are mean ± SEM from 8 mice. ^*^ *P* < 0.05 versus WT nonobstructed kidney.

Renal tubular damage signal triggers tissue remodeling and results in an increased accumulation of *α*-actin positive cells and collagen in injured tissues, leading to the progression of renal fibrosis [16]. We, therefore, examined whether sEH is a key player in renal interstitial fibrosis. Results of Masson's trichrome staining revealed that UUO surgery induced renal fibrosis in a time-dependent manner (Figure 5(a)). With Sirius Red Staining, we observed that UUO-induced accumulation of collagen in kidneys was attenuated by genetic disruption of* sEH *(Figure 5(b)). Moreover, western blotting results indicated that deletion of* sEH* ameliorates the protein expression of collagen 1A1 and *α*-actin in UUO kidneys as compared with that observed in WT mice (Figure 5(c)). In addition to interstitial fibrosis, chronic obstructive nephropathy causes the accumulation of type IV collagen accumulation at glomerular basement membrane, leading to the development of glomerulosclerosis [17]. We further delineated whether deletion of* sEH* affects the formation of glomerulosclerosis induced by UUO. After a 14-day UUO surgery, there were no significant differences in either the magnitude of glomerulosclerosis or the expression of type IV collagen in both WT and *sEH* ^−/−^ kidneys (Figures 6(a)–6(c)). Collectively, these findings suggest that sEH plays an important role in progression of interstitial fibrosis but not in glomerulosclerosis.

### 3.5. Ablation of sEH Alleviates UUO-Induced Increases in ROS Production and NADPH Oxidase Activity

**Figure 7:**
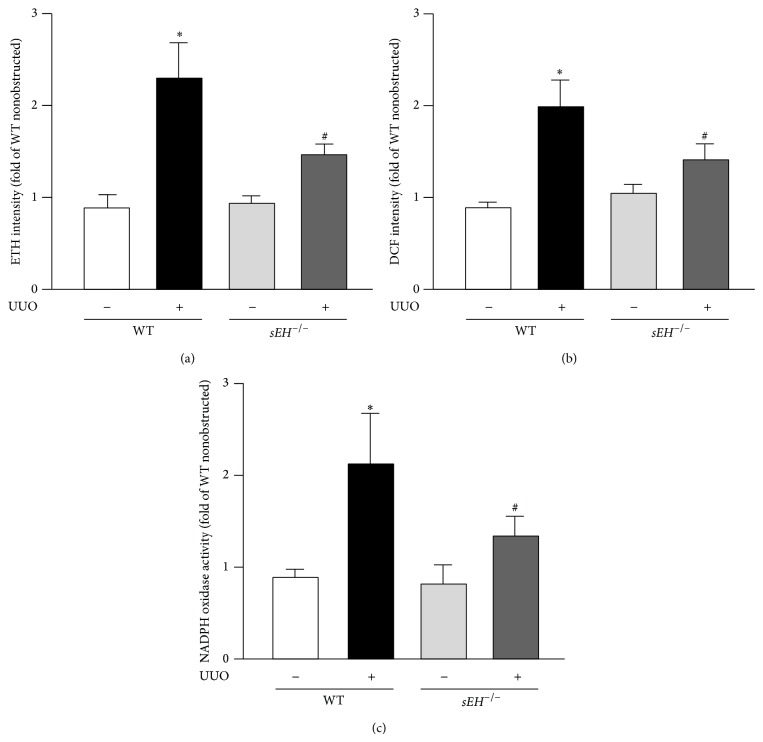
Deletion of* sEH* attenuates UUO-elicited increases in ROS production and NADPH oxidase activity. ((a) and (b)) Three days after UUO surgery, kidneys were harvested from WT and *sEH* ^−/−^ mice and subjected to measurement of O_2_^−^ and H_2_O_2_ by use of HE/ethidium and DCFH-DA/DCF assays. (c) NADPH oxidase activity of kidney lysates was analyzed by NADP+/NADPH assay kit. Data are mean ± SEM from 8 mice. ^*^ *P* < 0.05 versus WT nonobstructed kidney; ^#^ *P* < 0.05 versus WT UUO kidney.

Oxidative stress is reported to be highly associated with the regulation of inflammation [18]. We thus investigated whether O_2_^−^ and H_2_O_2_, 2 important ROS, were induced under UUO condition. Our results demonstrated that, with UUO surgery, ROS production was greater in WT mice than that in *sEH* ^−/−^ mice as revealed by DHE and DCFH-DA assays (Figures 7(a) and 7(b)). Similarly, UUO-induced increase in NADPH oxidase activity was decreased in *sEH* ^−/−^ mice as compared to that observed in WT mice (Figure 7(c)).

## 4. Discussion

The biological significance of sEH in renal physiology and pathology has not been fully elucidated. In this study, we identified the important role of sEH in the pathogenesis of obstructive nephropathy with a mouse UUO model. We demonstrated that genetic disruption of* sEH* ameliorates hydronephrosis, renal tubular injury, inflammation, and fibrosis induced by UUO. Interestingly, our results indicated that deletion of* sEH* did not influence the development of glomerulosclerosis, which is a common feature of chronic kidney disease and subsequent renal dysfunction; this result was consistent with the previous findings by Jung et al. that sEH inhibition does not prevent progression of renal glomerulosclerosis in the progressive renal disease model [19]. Additionally,* sEH *deficiency profoundly decreased macrophage infiltration in UUO kidneys, which might be attributed to the downregulation of the inflammatory mediators in UUO-induced nephropathy. Therefore, sEH may be a positive regulator of inflammation in the progression of experimental obstructive nephropathy.

Notably, we also found that sEH-positive signals in the inflamed areas of UUO kidneys were mainly localized in macrophages, which was consistent with recent findings that functional loss of sEH attenuated macrophage-mediated inflammation [20]. Our results further demonstrated that genetic deletion of* sEH* did not change the infiltration of neutrophils and lymphocytes as evident by the protein level of MPO and CD3 in kidney with UUO surgery. These findings suggest that sEH might be also implicated in the regulation of macrophage-mediated immunity in UUO-induced nephropathy. Growing evidence suggests that increasing the accumulated levels of EETs in inflamed tissues by inhibiting sEH with specific pharmacological inhibitors can be an effective therapeutic strategy in treating inflammatory diseases [21]. Mechanically, inhibition of sEH or treatment with EETs can reduce the cytokine- or chemokine-mediated chemotaxis and prevent leukocyte infiltration into inflamed tissues [22, 23], which are in agreement with our current findings: the production of proinflammatory mediators including MCP-1, MIP-2, IL-1*β*, and IL-6, as well as iNOS, induced by UUO was decreased in* sEH* ^−/−^ mice as compared to UUO-treated WT mice. All of these mediators are known to be heavily involved in the biology of macrophages in inflammation [24, 25]. Very recently, an elegant study by Kim et al. reported that, in a UUO model, NF-*κ*B activation as one of inflammatory responses was attenuated by sEH inhibition [25]. Collectively, these findings are in line with the previous reports that sEH plays a crucial role in regulating the inflammatory response of UUO-induced renal injury and may represent a potential therapeutic target in inflammatory diseases [12, 26].

A role of the hydrolase activity of sEH in the metabolism of EETs, inflammation, and hypertension has been well documented. Pharmacological inhibition of hydrolase activity of sEH or genetic deletion of* sEH* leads to the accumulation of EETs in tissues and plasma and thus enhances the physiological function of cardiovascular system and impedes the development of cardiovascular diseases and inflammatory diseases. sEH is abundantly expressed in the kidney, implying that it might play an important role in regulating the pathophysiological function in kidney. Indeed, growing evidence suggests that inhibition of sEH provides protection against kidney associated diseases in experiment models [27]. For instance, inhibition of sEH decreases renal inflammation and improves the renal function in hypertension- or diabetes-induced renal injury [9, 11]. However, the information about the role of sEH and its potential molecular mechanism in renal fibrosis is still limited. Here, we showed that genetic ablation of* sEH* lessened the UUO-induced interstitial fibrosis as evidence by the decrease in the levels of collagen and *α*-actin, which is consistent with the very recent findings by Kim et al. that inhibition of sEH by pharmacological inhibitor trans-4-{4-[3-(4-trifluoromethoxy-phenyl)-ureido]-cyclohexyloxy} benzoic acid or genetic deletion in mice prevents the renal inflammation and fibrosis [25, 28]. Collectively, these findings suggest that inhibition of sEH may have potential therapeutic value in treating fibrotic diseases.

Leukocyte infiltration and cytokine production as indicators of inflammatory status are crucial in determining the stages of certain inflammatory diseases [14, 24]. These key inflammatory events can trigger the process of tissue remodeling and fibrosis such as cell proliferation and apoptosis, epithelial-mesenchymal transition (MET), fibroblast activation, and extracellular matrix deposition [16, 17]. Several lines of evidence indicate that inhibition of sEH activity provides anti-inflammatory action and thus limits the development of arteriovenous graft stenosis, inflammatory bowel disease, and hepatic steatosis [19, 22, 29]. In addition, Sander et al. reported that treatment with EETs or ablation of* sEH* activity stimulates the process of wound healing in experimental animals [30, 31]. Moreover, Kompa et al. demonstrated that inhibition of sEH reduces collagen synthesis of cardiac fibroblasts and leads to the alleviation of cardiac fibrosis in the postmyocardial infarction mouse model [10]. On the contrary, Wang et al. showed that overexpression of* sEH* promotes EMT in rat proximal tubular epithelial cells [32]. In agreement with the findings by Sander et al., Zhao et al. reported that overexpression of human CYP2J2, which metabolizes arachidonic acid into EETs, decreases expression of type I and IV collagen and thereby retards the renal fibrosis in 5/6 nephrectomized rats [33]. This notion was further supported by our findings in this study that genetic disruption of* sEH* decreased renal inflammation and led to attenuation of renal fibrosis. Taken together, these findings strongly suggest that sEH might play a key role in progression of tissue fibrosis. However, further investigation is still required to delineate how sEH regulates the cellular and molecular mechanisms underlying fibrosis.

Importantly, oxidative stress is known to be highly associated with the regulation of inflammation in a variety of inflammatory diseases [14, 18]. This notion was further supported by our findings that the levels of superoxide anion radical and hydrogen peroxide in UUO kidneys were increased in WT mice, which was reduced in UUO kidneys of* sEH* ^−/−^ mice. These lines of evidence imply that sEH may have prooxidative and proinflammatory properties. Collectively, these observations strongly suggest the heavy involvement of sEH in the regulation of kidney physiologic function and the development of kidney diseases.

## 5. Conclusions

In conclusion, we demonstrate the role of sEH in progression of obstructive nephropathy. Deletion of* sEH* gene ameliorates the renal injury by decreasing inflammation, renal tubular injury, and renal interstitial fibrosis. The mechanisms revealed in this study provide novel insights for delineating the role of sEH in inflammatory diseases. Our findings suggest that targeting sEH may have clinical implications and may be a valuable therapeutic strategy in treating obstructive nephropathy-related kidney diseases.

## Figures and Tables

**Table 1:** Microscopy assessment of histological changes in UUO-induced renal injury.

	0	1	2	3	4	5
Tubular dilation	Normal	<10%	10–25%	26–50%	51–75%	>75%
Tubular atrophy	Normal	<10%	10–25%	26–50%	51–75%	>75%
Leukocyte infiltration	Absent	<10%	10–25%	26–50%	51–75%	>75%
Interstitial volume	Normal	<10%	10–25%	26–50%	51–75%	>75%

**Table 2:** Microscopy assessment of histological changes in UUO-induced glomerulosclerosis.

Score	Degree of sclerosis
0	Normal glomerulus
1	25%
2	25–50%
3	50–75%
4	>75%
